# Association between air pollution and acute exacerbation of chronic obstructive pulmonary disease in Hebei Province: A time-series study

**DOI:** 10.1097/MD.0000000000050080

**Published:** 2026-08-14

**Authors:** Huixia Shi, Jiajun Li, Boyan Li, Dan Li, Yatian Liu, Xinrui Li, Jie Liu, Longmei Tang

**Affiliations:** a School of Public Health, Hebei Medical University, Shijiazhuang, China; b Shijiazhuang Center for Disease Control and Prevention, Shijiazhuang, China; c Hebei Key Laboratory of Intractable Pathogens, Shijiazhuang Center for Disease Control and Prevention, Shijiazhuang, Hebei, China; d Senior Department of Cardiology, The Sixth Medical Center of PLA General Hospital, Beijing, China; e Hebei Key Laboratory of Environment and Human Health, Shijiazhuang, China.

**Keywords:** acute exacerbations of chronic obstructive pulmonary disease (AECOPD), air pollution levels, distributed lag nonlinear model (DLNM), multivariate meta-analysis, relative risk (RR), time-series analysis

## Abstract

Evidence regarding the short-term effects of ambient air pollution on acute exacerbations of chronic obstructive pulmonary disease (AECOPD) across multiple cities in northern China remains limited. This study aimed to evaluate the associations between major ambient air pollutants and AECOPD hospitalizations, as well as their lagged effects. We conducted an ecological time-series study of 14,786 AECOPD hospitalizations recorded at 49 hospitals in Shijiazhuang, Handan, and Chengde, Hebei Province, from January 1, 2018, to December 31, 2020. City-specific associations between daily concentrations of fine particulate matter (PM_2_._5_), inhalable particulate matter (PM_10_), sulfur dioxide (SO_2_), nitrogen dioxide (NO_2_), carbon monoxide (CO), and 8-hour ozone and AECOPD hospitalizations were estimated using distributed lag nonlinear models, with adjustment for long-term trends, temperature, relative humidity, day of the week, public holidays, and stages of the coronavirus disease 2019 pandemic. Single-day lag effects from lag 0 to lag 5, cumulative lag effects from lag 0–1 to lag 0–5, effects of extreme pollutant concentrations, and associations stratified by age and sex were evaluated. City-specific estimates were subsequently pooled using random-effects multivariate meta-analysis. Associations were expressed as relative risks with 95% confidence intervals. During the 1096-day study period, 14,786 AECOPD hospitalizations were recorded. Compared with the 25th-percentile concentrations, the 75th-percentile concentrations of PM_2.5_, PM_10_, SO_2_, NO_2_, and CO generally showed stronger cumulative associations with longer lag periods, with the largest estimates usually observed at lag 0–5 days, whereas single-day effects were weak. All 6 pollutants exhibited nonlinear cumulative exposure–response relationships. Compared with median concentrations, the 99th-percentile concentrations of PM_2.5_, PM_10_, SO_2_, NO_2_, and CO were associated with higher hospitalization risks. High PM_2.5_ reached its maximum effect at lag 0–5 days (relative risk, 1.391; 95% confidence interval, 1.169–1.658). Higher 8-hour ozone concentrations were generally associated with lower hospitalization risk. Associations were more pronounced among adults aged ≥65 years and males. Short-term exposure to PM_2_._5_, PM_10_, SO_2_, NO_2_, and CO was associated with increased AECOPD hospitalization risk, with nonlinear, lagged, and cumulative effects. Older adults and females may be more susceptible to ambient air pollution.

## 1. Introduction

Chronic obstructive pulmonary disease (COPD) is characterized by persistent airflow limitation with progressive development and is associated with an enhanced chronic inflammatory response of the airways and lung tissues to noxious particles or gases.^[1]^ COPD was the 4th leading cause of death globally in 2021,^[2]^ and it is projected to become the 5th leading cause of global disease burden and the 3rd leading cause of death by 2030.^[3]^ In recent years, although the mortality rate of COPD in China has declined, it remains higher than the global average, with COPD-related deaths in China accounting for more than 30% of the worldwide total.^[4]^

Acute exacerbation of chronic obstructive pulmonary disease (AECOPD) is a key event in the management of COPD and is typically characterized by an acute worsening of respiratory symptoms accompanied by pulmonary infection.^[5]^ AECOPD can lead to accelerated decline in lung function, reduced quality of life, and increased mortality.^[6–8]^ Previous studies have demonstrated that deaths among patients with AECOPD frequently occur within a short period after hospitalization.^[9,10]^ Within healthcare systems, hospitalizations and emergency treatments related to AECOPD account for the largest proportion of the overall disease burden of COPD.^[11]^

Air pollution is an important environmental risk factor affecting respiratory health.^[12]^ With the rapid progression of industrialization and urbanization, air pollution has become a growing global concern. Previous studies have demonstrated that pollutants such as particulate matter (PM_2_._5_ and PM_10_), nitrogen dioxide (NO_2_), sulfur dioxide (SO_2_), and ozone (O_3_) can aggravate respiratory injury and increase the risk of acute exacerbation of COPD through mechanisms including oxidative stress, airway inflammation, and immune dysfunction.^[13,14]^ Multiple international time-series and case-crossover studies have confirmed that short-term exposure to air pollution is significantly associated with increased risks of outpatient visits, emergency department visits, and hospitalizations for AECOPD.^[15,16]^ Studies conducted in the United States, South Korea, and South America have consistently reported that elevated PM concentrations are associated with an increased risk of AECOPD, and this association persists even in regions with relatively good air quality.^[16–18]^

In recent years, studies on the relationship between air pollution and AECOPD in China have gradually increased; however, most have been limited to single cities, such as Beijing, Shanghai, Jinan, and Changchun, resulting in relatively restricted study areas. Due to differences in climatic conditions, topographical characteristics, energy structures, and pollutant compositions across regions, the findings of these studies have shown a certain degree of heterogeneity.^[19–22]^ In addition, studies investigating the association between air pollution and AECOPD across multiple cities in North China, particularly in Hebei Province, remain limited. Hebei Province, located in the North China Plain, is characterized by concentrated industrial cities, high energy consumption, and complex terrain, making its air pollution patterns regionally representative. Therefore, regional studies are warranted to further clarify the short-term health effects of air pollution on AECOPD.

In this study, hospitalization data for AECOPD from three cities in Hebei Province during 2018–2020 were collected, together with concentrations of 6 major ambient air pollutants during the same period. A time-series analysis was conducted to evaluate the association between air pollution exposure and the risk of AECOPD hospitalization, and the lag effect characteristics of different pollutants were further investigated. The findings of this study may provide scientific evidence for health risk assessment of air pollution and the development of COPD prevention and control strategies in North China, while also offering references for comparative studies on the health effects of air pollution across different regions in China.

## 2. Materials and methods

### 2.1. Study setting

In this study, Shijiazhuang, Handan, and Chengde cities were selected from 11 prefecture-level cities of Hebei province. Shijiazhuang, as the capital of Hebei Province, is located in the south-central part of Hebei Province; Handan is located at the southernmost end of Hebei Province; and Chengde is located in the connection transition zone between North China and Northeast China, and the northeast of Hebei Province. The above-mentioned regions have historically been characterized by relatively high PM_2_._5_ concentrations, with substantial contributions from industrial emissions and regional pollutant transport.^[23,24]^

### 2.2. Data sources

The AECOPD hospitalization data were selected from a total of 49 hospitals in 3 cities in Hebei Province that participated in health cost accounting in Hebei Province for 3 consecutive years from 2018 to 2020, and all patient information in the inpatient cost settlement system was exported. Data information included gender, date of birth, date of hospitalization, date of discharge, primary diagnosis, secondary diagnosis, and International Classification of Diseases, 10th Edition (ICD-10) coding.^[25]^ Cases with missing ICD-10 codes were recode according to the primary diagnosis. Cases were included in this study according to ICD-10 code J44.100 (primary diagnosis of acute exacerbations of COPD). We enrolled a total of 14786 AECOPD hospitalizations in Hebei Province, including 4353 cases in Shijiazhuang, 3176 cases in Handan, and 7257 cases in Chengde.

Pollution data were obtained from the air quality online monitoring and analysis platform (http://www.aqistudy.cn), including PM_2.5_, PM_10_, SO_2_, NO_2_, carbon monoxide (CO), 8-hour ozone concentration (O_3__8h). Meteorological data were obtained from the National Meteorological Information Center (https://data.cma.cn), including temperature, humidity, and wind level.

Missing value processing: meteorological variables contained 13 missing observations (1.19% of all daily records) during the study period (2018–2020). Missing values were imputed using the mean of the values from the preceding and following days within the same period. No missing data were observed for air pollutant concentrations.

This study was reviewed and approved by the Medical Ethics Committee of Hebei Medical University (No. 2024047). This ecological time-series study involved the secondary analysis of existing de-identified and aggregated hospitalization data, with no direct contact with patients. Individual informed consent was therefore not required.

### 2.3. Statistical analysis

#### 2.3.1. Descriptive statistics methods

The daily number of AECOPD hospitalizations and the number of hospitalizations with different demographic characteristics (gender, age) from January 1, 2018 to December 31, 2020 were described, and the distribution characteristics of each pollutant concentration (PM_2.5_, PM_10_, SO_2_, NO_2_, CO, O_3__8h) were analyzed.

#### 2.3.2. Construction of statistical models

In this study, a 2-stage analysis was used to model the effect of air pollutant levels on AECOPD.

In the 1st stage, we estimated the association between air pollutant levels and the number of hospitalizations for AECOPD using a time-series design. For the general population, AECOPD daily hospitalization data represent a small probability event, and the actual distribution approximates a Poisson distribution. Therefore, this study employed distributed lag nonlinear models to reflect the relationship between daily pollutant concentrations and the number of hospitalizations for acute exacerbations of COPD.^[26]^ We used natural spline functions to control for potential confounding factors, including long-term time trends, relative humidity, temperature, day of the week, holidays, and the impact of the coronavirus disease 2019 (COVID-19) pandemic.^[18,27]^ Considering that the COVID-19 pandemic may have substantially affected healthcare utilization and hospitalization patterns, the pandemic period was further adjusted as a time-varying categorical variable. According to official COVID-19 prevention and control policies issued by the Hebei Provincial Government during the COVID-19 pandemic, the study period was classified into 4 stages: non-pandemic period (January 1, 2018–January 23, 2020), first-level emergency response period (January 24, 2020–May 1, 2020), second-level emergency response period (May 2, 2020–July 10, 2020), and routine prevention and control period (July 11, 2020–December 31, 2020). This classification approach was intended to better capture the dynamic influence of epidemic prevention and control measures on AECOPD hospitalizations.

In this study, 5 degrees of freedom per year were used for the time-smoothing function to control for long-term trends in hospitalization counts, while 3 degrees of freedom were assigned to daily mean temperature and relative humidity. The selection of degrees of freedom was based on previous time-series studies of air pollution.^[28–30]^ Thus, the final city-specific model is as follows.


$$\begin{array}{c} \begin{array}{cc} & E(Yt)=\alpha +\beta_{i}Z_{t}+ns(time,df=8/per\;year)\\ & +ns(temp,df=3)+ns(rhum,df=3)\\ & +\kappa holiday_{t}+\xi COVID-19t+\eta dow_{t} \end{array} \end{array}$$

Where *Yt* is the number of AECOPD hospitalizations on day *t*; *E*(*Yt*) refers to the expected number of AECOPD hospitalizations on lag day *t*; and *Z*_*t*_ is a cross-basis function of air pollutant levels. *α* is the intercept; *β*_*i*_ is the regression coefficient; *ns* is the natural spline smoothing function; *temp* is the mean temperature on day *t*; *time* is the date variable; *rhum* is the mean relative humidity on day *t*; $\eta dow_{t}$ refers to the week effect, $\kappa holiday_{t}$ refers to the holiday effect, which is a phenomenon of periodic fluctuations in the number of hospital visits due to weekends and holidays; and ξCOVID−19t is the fixed effect of the new coronavirus epidemic.

To assess the short-term and delayed effects of air pollutant exposure on AECOPD hospitalizations, we estimated single-day lag effects from lag 0 to lag 5 and cumulative lag effects from lag 0–1 to lag 0–5. Based on previous studies of short-term air pollution exposure and AECOPD hospitalizations, and considering that the acute respiratory effects of air pollutants generally occur within several days after exposure, the maximum lag period was set at 5 days.^[31,32]^ For the primary lag-effect analysis, the 25th-percentile concentration (P25) of each pollutant was used as the reference, and the relative risk (RR) at the 75th-percentile concentration (P75) was estimated for each single-day and cumulative lag period. In addition, the overall cumulative exposure–response relationship was evaluated over lag 0–5 days, using the median concentration of each pollutant as the reference. The single-day and cumulative effects of extremely low (P1) and extremely high (P99) pollutant concentrations were also evaluated relative to the corresponding median concentration. Finally, subgroup analyses were conducted by age (<65 and ≥65 years) and sex (male and female) to assess potential differences in susceptibility. Effect estimates were expressed as RRs with 95% confidence intervals (CIs).

In the 2nd stage, random-effects multivariate meta-analysis was used to estimate the combined effects of different air pollution levels on the number of hospitalizations for AECOPD in different cities.^[33]^ The model can be described as follows:


$$\begin{array}{c} \hat{\theta_{i}}\sim N_{k}\left(\theta ,S_{i}+\psi \right) \end{array}$$

where *θ*_*i*_ is the true unrecognized *k*-length vector of parameters for studies *i. θ*_*i*_, subjected to the *k*-dimensional multivariate normal distribution *N*_*k*_(*θ, S*_*i*_), is the estimated regression coefficient of *θ*_*i*_ that is derived from the 1st-stage model. *S*_*i*_ and *ψ* represent the within-study and between-study variance–covariance matrices, respectively.

#### 2.3.3. Sensitivity analysis

To test the robustness of the results, sensitivity analysis was performed by changing the degrees of freedom (*df*) from 2 to 4 for meteorological factors and from 4 to 6 for long-term trend time.

All analyses were performed using R software version 4.0.5 with the packages “dlnm,” “splines,” “ggplot2,” and “mvmeta.”

## 3. Results

### 3.1. Descriptive statistical analysis

From January 01, 2018 to December 31, 2020 (1096 days total), 14,786 hospitalizations were recorded. The characteristics of the daily number of AECOPD hospitalizations and air pollutant levels were shown in Table 1.

**Table 1:** Summary statistics of AECOPD and air pollutant levels in Hebei Province.

Variables	Min	P_1_	P_25_	P_50_	P_75_	P_99_	Max	IQR
Daily hospitalizations								
Total	0	0	2	4	6	16	25	4
Sex group								
Males	0	0	1	2	4	10	15	3
Females	0	0	0	1	2	7	14	2
Age group								
<65	0	0	0	1	2	5	8	2
≥65	0	0	2	3	5	12	19	3
Atmospheric pollutant levels								
PM_2.5_ (µg/m^3^)	6	9	24	38	61	232	354	37
PM_10_ (µg/m^3^)	11	18	54	83	123	343	490	69
SO_2_ (µg/m^3^)	2	4	8	12	18	54	86	10
NO_2_ (µg/m^3^)	5	10	24	34	48	93	122	24
CO (mg/m^3^)	0.2	0.3	0.6	0.8	1.2	3.1	4.9	0.6
O_3__8h (µg/m^3^)	4	10	58	93	140	241	310	52

CO = carbon monoxide, IQR = interquartile range, NO_2_ = nitrogen dioxide, O_3__8h = 8-hour ozone concentration, P_1_ = 1st percentile, P_25_ = 25th percentile, P_50_ = median, P_75_ = 75th percentile, P_99_ = 99th percentile, PM_10_ = inhalable particulate matter, PM_2.5_ = fine particulate matter, SO_2_ = sulfur dioxide.

### 3.2. Single and cumulative lag effects of P75 versus P25 pollutant concentrations

**Figure 1.**
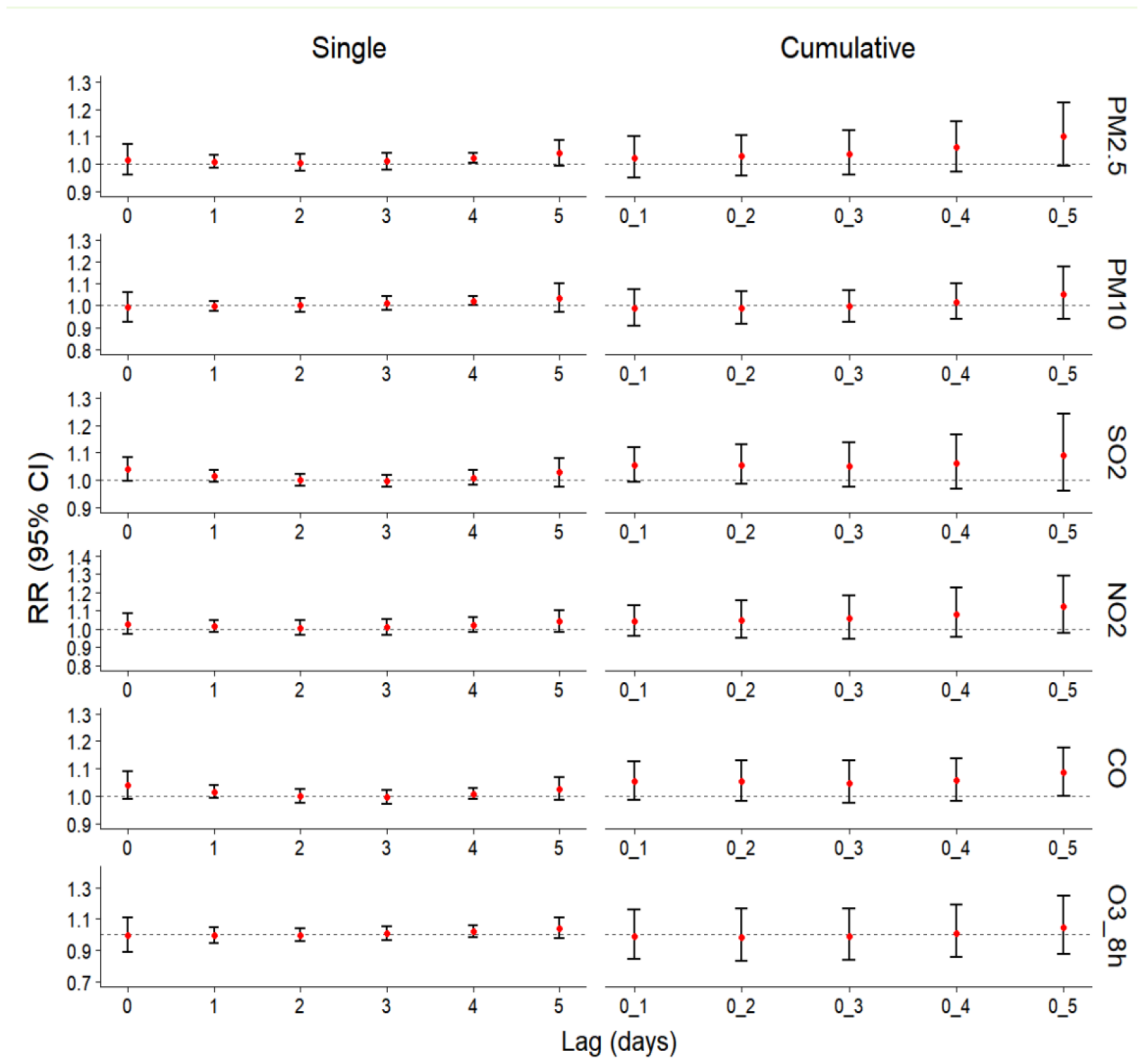
Single-day and cumulative lag effects of air pollutant concentrations at the 75th percentile relative to the 25th percentile on daily AECOPD hospitalizations. AECOPD = acute exacerbation of chronic obstructive pulmonary disease.

We use distributed lag nonlinear models to estimate the single-day and cumulative lag effects of air pollutant concentrations at the 75th percentile (P75) relative to the 25th percentile (P25) on AECOPD hospitalizations, as shown in Figure 1. After adjustment for long-term trends, temperature, relative humidity, day of the week, public holidays, and stages of the COVID-19 pandemic, the single-day effect estimates for all six pollutants were generally close to 1.0. For PM_2.5_, PM_10_, SO_2_, NO_2_, and CO, the cumulative RRs showed a modest increasing trend with longer lag periods, with the largest effect estimates generally observed at lag 0–5 days. This increasing cumulative pattern was particularly evident for PM_2.5_, NO_2_, and CO, whereas the cumulative effect estimates for SO_2_ remained relatively stable across lag periods. In contrast, the single-day and cumulative effect estimates for O_3__8h fluctuated around 1.0.

### 3.3. Cumulative effect of air pollutant levels on the number of AECOPD hospitalizations within 5 days

**Figure 2.**
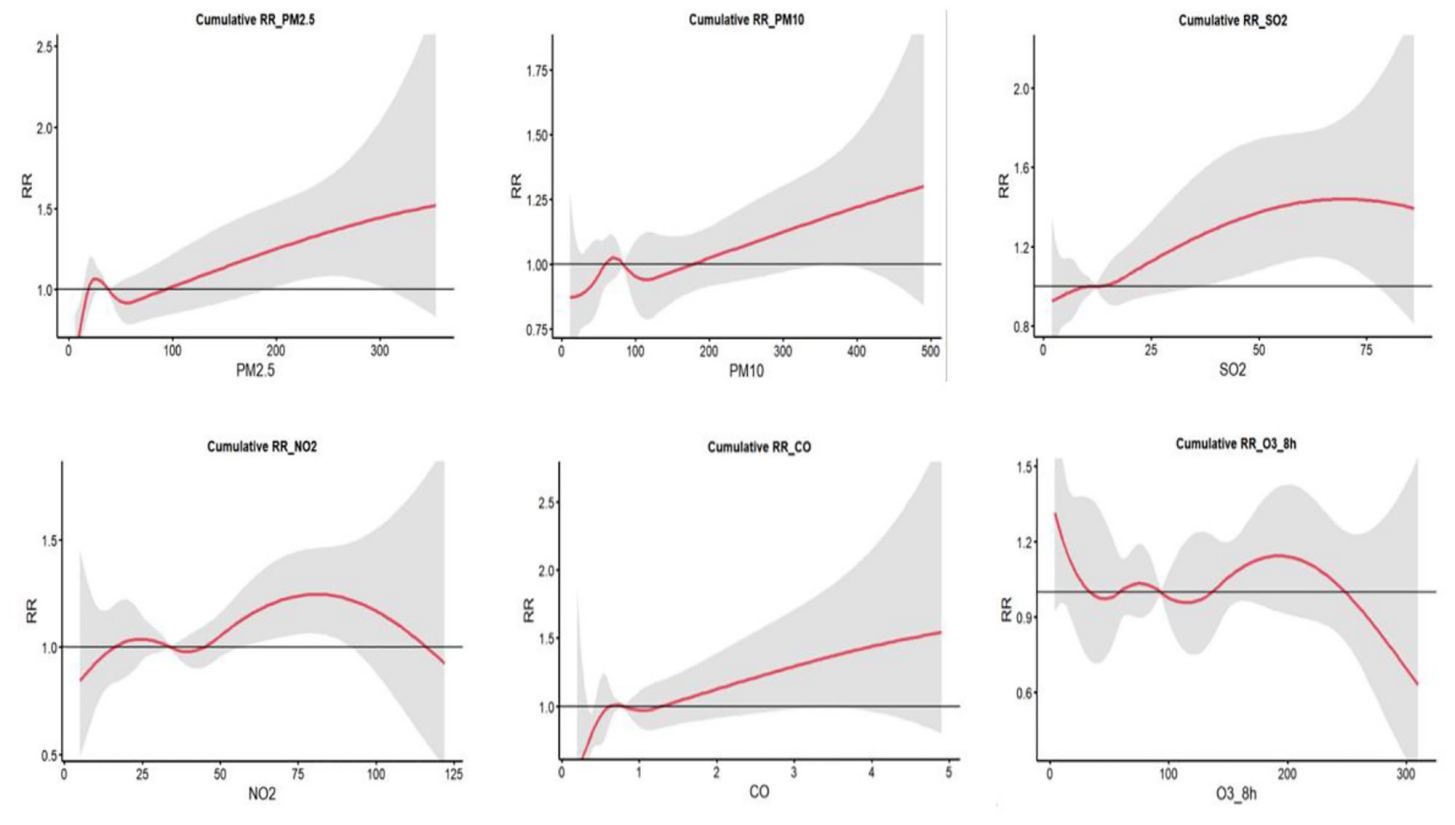
Cumulative effect of air pollutant levels on the number of daily hospitalizations for AECOPD within 5 days (compared to the 50th percentile of air pollutant levels). AECOPD = acute exacerbation of chronic obstructive pulmonary disease.

Within the lag 0–5-day period, all 6 air pollutants exhibited nonlinear cumulative exposure–response relationships with AECOPD hospitalizations (Fig. 2). For PM_2.5_, the cumulative RR increased sharply at lower concentrations, subsequently declined, and then increased monotonically thereafter. PM_10_ showed a similar pattern, with an initial fluctuation in RR followed by a sustained monotonic increase as concentrations rose. The cumulative RR for SO_2_ increased progressively with increasing concentrations, plateaued near the upper end of the exposure range, and then declined slightly. For NO_2_, the RR initially increased, decreased modestly at intermediate concentrations, subsequently increased again, and then declined at higher concentrations. Overall, CO showed a gradual increase in RR with increasing exposure concentrations. In contrast, the exposure–response curve for O_3__8h exhibited more pronounced fluctuations: the RR initially decreased, fluctuated around the reference level at low-to-moderate concentrations, and then declined markedly at higher concentrations.

### 3.4. Single and cumulative lagged effects of extreme pollutant concentrations in air pollutant levels on the number of daily hospitalizations for AECOPD

**Figure 3.**
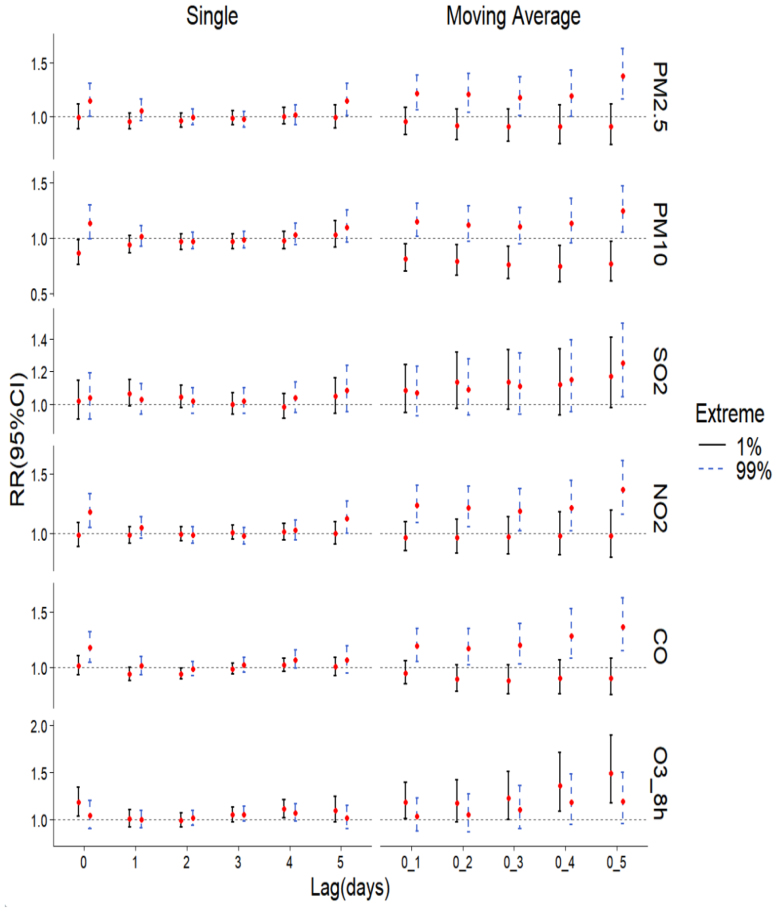
Single and cumulative lagged effects of extreme pollutant concentrations in air pollutant levels on the number of daily hospitalizations for AECOPD (compared to the 50th percentile of air pollutant levels). AECOPD = acute exacerbation of chronic obstructive pulmonary disease.

Figure 3 illustrates the single and cumulative lag effects of extreme air pollutant concentrations on AECOPD hospitalizations, using the median concentration of each pollutant as the reference value. Overall, higher concentrations (99th percentile) of PM_2.5_, PM_10_, SO_2_, NO_2_, and CO were associated with increased hospitalization risk, and these associations became more pronounced under cumulative lag structures, with RR generally increasing across longer lag days. In contrast, lower concentrations (1st percentile) of PM_2.5_, PM_10_, NO_2_, and CO were associated with lower hospitalization risk.

For single-day lag effects, the highest RRs for high concentrations of PM_2.5_, PM_10_, SO_2_, and CO were generally observed at lag 0, although statistical significance was not observed for SO_2_. High NO_2_ concentrations also showed a significant effect at lag 0, with an RR of 1.183 (95% CI: 1.069–1.310).

Under the cumulative lag structure, the association between high PM_2.5_ concentrations and AECOPD hospitalizations gradually strengthened as the lag period increased, reaching the highest RR at lag 0–5 days (RR = 1.391, 95% CI: 1.169–1.658). In contrast, low PM_2.5_ concentrations were associated with a progressively lower hospitalization risk over longer cumulative lag periods. Similar cumulative patterns were observed for NO_2_ and CO. Overall, higher O_3_ concentrations were generally associated with a lower risk of AECOPD hospitalization; however, the magnitude of the associations between both high and low O_3_ concentrations and hospitalization risk increased with longer cumulative lag periods.

### 3.5. Subgroup analysis

To investigate potential effect modification by age and sex, stratified analyses were conducted by dividing participants into older (≥65 years) and younger (<65 years) groups, as well as male and female groups. Subgroup analyses demonstrated heterogeneity in susceptibility to air pollution exposure across different populations.

**Figure 4.**
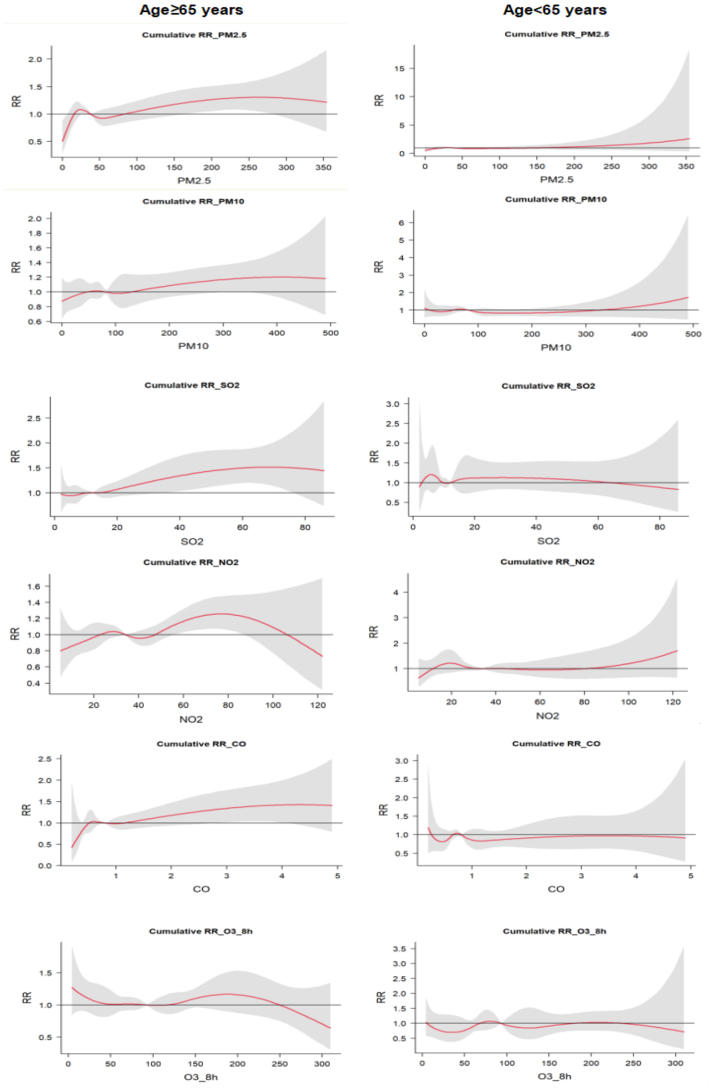
Effect of air pollutant levels on the number of daily hospitalizations for AECOPD in different age groups (compared to the 50th percentile of air pollutant levels). AECOPD = acute exacerbation of chronic obstructive pulmonary disease.

In the age-stratified analysis (Fig. 4), PM_2.5_, PM_10_, SO_2_, and NO_2_ showed stronger positive exposure–response relationships among individuals aged ≥65 years, whereas these associations were weaker or less apparent among individuals aged <65 years. Older adults also appeared to be more sensitive to CO and O_3_ exposure. Elevated CO concentrations were associated with increased hospitalization risk among individuals aged ≥65 years. In addition, lower O_3_ concentrations were associated with increased hospitalization risk in the older population.

**Figure 5.**
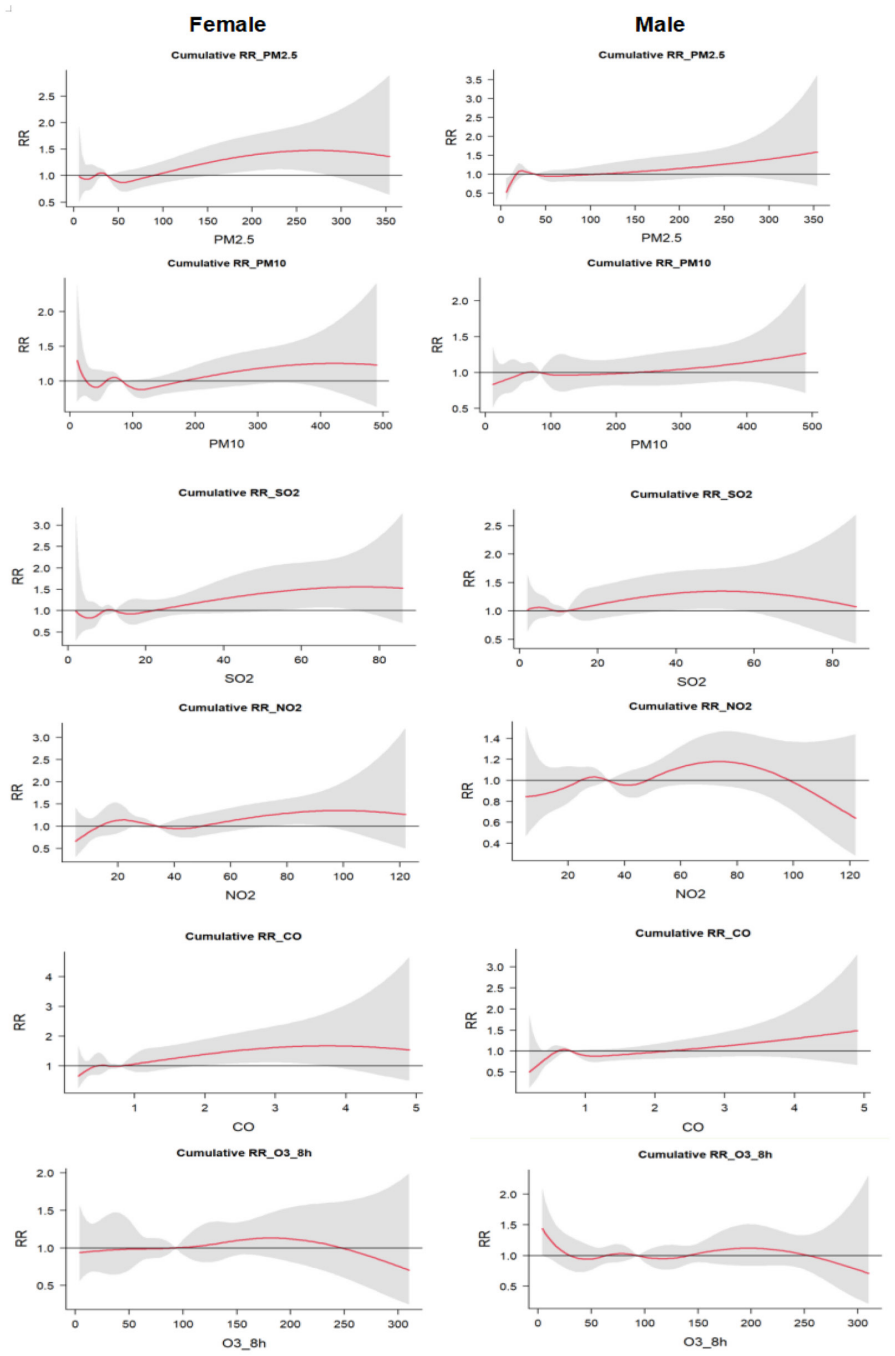
Effect of air pollutant levels on the number of daily hospitalizations for AECOPD in different sex groups (compared to the 50th percentile of air pollutant levels). AECOPD = acute exacerbation of chronic obstructive pulmonary disease.

In the sex-stratified analysis (Fig. 5), PM_2.5_, PM_10_, and CO showed relatively stronger positive associations among females, whereas O_3__8h appeared to show stronger associations among males, particularly at lower concentrations. SO_2_ and NO_2_ were positively associated with AECOPD hospitalizations in both sexes, although the exposure–response patterns differed between males and females.

### 3.6. Sensitivity analysis

**Figure 6.**
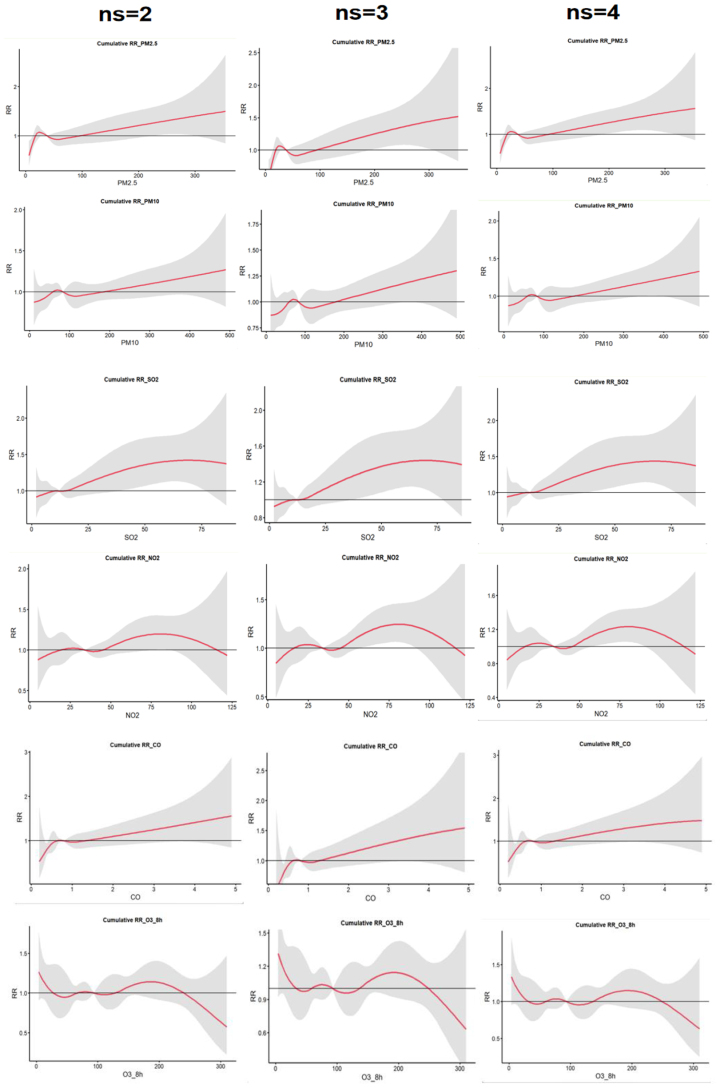
Effect of changing degrees of freedom of meteorological factors (2–4) and air pollutant levels on the daily number of hospitalizations for AECOPD (compared to the 50th percentile of air pollutant levels). AECOPD = acute exacerbation of chronic obstructive pulmonary disease.

**Figure 7.**
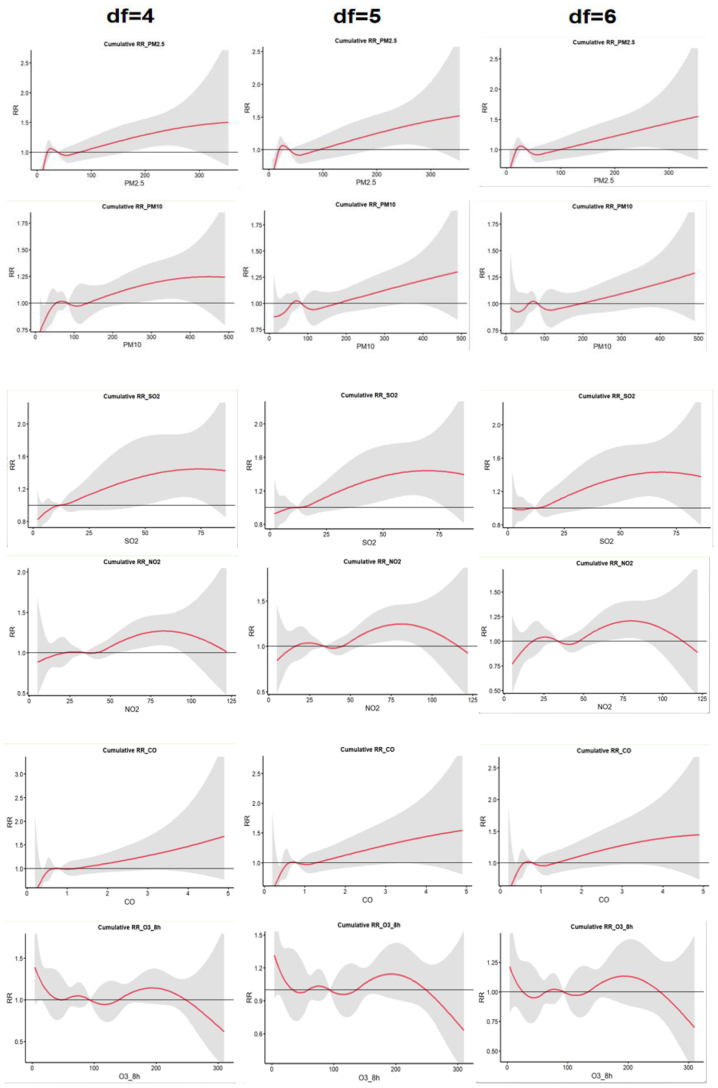
Effect of changing degrees of freedom of long-term trend (*time*) (4–6) and air pollutant levels on the daily number of hospitalizations for AECOPD (compared to the 50th percentile of air pollutant levels). AECOPD = acute exacerbation of chronic obstructive pulmonary disease.

The results of the sensitivity analysis are shown in Figures 6 and 7. Although the RR values changed with different degrees of freedom of the natural spline function (*ns* = 2, 3, 4) or time trend (*time*) freedom (*df* = 4, 5, 6), the overall effect trend did not change, confirming the robustness of the current model for estimating the exposure–response relation of air pollutant level factors on the number of AECOPD hospitalizations.

## 4. Discussion

The increasing trend of air pollutant levels, especially in developing countries, has been consistently associated with a range of adverse health outcomes. In this study, we found that increasing concentrations of PM_2.5_, PM_10_, SO_2_, NO_2_, and CO resulted in an increase in the number of AECOPD hospitalizations, while increasing concentrations of O_3__8h decreased the risk of AECOPD. Overall, these findings are in agreement with most previous epidemiological studies, which have demonstrated that PM and certain gaseous pollutants increase the risk of acute exacerbations of COPD.^[16,19,32]^

Previous studies have shown that PM_2.5_ and PM_10_ can induce oxidative stress and inflammatory responses, damage airway epithelial cells, and impair immune function, thereby promoting acute exacerbations of COPD.^[34,35]^ Some evidence further suggests that PM_2.5_ may exacerbate airway inflammation by increasing reactive oxygen species production and causing mitochondrial dysfunction.^[36,37]^ In the present study, PM exhibited cumulative lag effects, suggesting that it may exert its effects through sustained inflammatory processes. SO_2_ and NO_2_, as irritant gases, can induce airway inflammation and oxidative damage, thereby increasing the burden on the respiratory system.^[38]^ Elevated CO concentrations have been associated with an increased risk of AECOPD, which may be related to tissue hypoxia and cytotoxic effects.^[39]^

It is worth noting that in this study, low concentrations of CO appeared to be associated with a reduced risk of AECOPD, and O_3__8h showed an inverse association with outcomes, which is inconsistent with findings from some previous studies.^[19,40]^ This discrepancy may be influenced by regional pollution profiles, intercorrelations among pollutants, and lag structure specifications. On the one hand, experimental evidence has suggested that low-dose CO may exert anti-inflammatory or cytoprotective effects,^[41]^ although population-level evidence remains limited. On the other hand, these findings may also reflect residual confounding, exposure misclassification, or bias introduced by model specification. Therefore, the apparent “protective effect” of ozone may also be related to differences in study populations, insufficient sample size, or variations in study design, rather than representing a true biological protective mechanism.

Subgroup analyses indicated that older adults (≥65 years) and females were more sensitive to air pollution exposure. The increased vulnerability among older individuals may be attributed to impaired respiratory defense mechanisms and a higher burden of comorbidities.^[42]^ Greater susceptibility in females has also been reported in some previous studies,^[19,43]^ however, the underlying biological mechanisms remain to be further elucidated.

## 5. Limitations

This study analyzed the effects of atmospheric pollutant levels on the number of AECOPD hospitalizations and their lagged effects, providing a scientific basis for the relevant departments to take effective intervention measures and set reasonable concentration limits, but there are still some shortcomings in the study. First, this study was an ecological time-series study that used city-level average air pollutant concentrations instead of individual exposure levels. This approach could not fully capture within-city spatial heterogeneity or individual exposure differences, which may have resulted in exposure misclassification and measurement error, thereby reducing the precision of the model estimates. In addition, the study period spanned from 2018 to 2020, during which the COVID-19 pandemic may have affected healthcare utilization and hospitalization patterns. Although the COVID-19 pandemic was adjusted in the model as a categorical variable according to the strictness of prevention and control measures, its potential influence could not be completely eliminated. Finally, this study included data from only 3 cities in Hebei Province. Although Hebei Province is characterized by complex terrain and a relatively representative industrial structure of northern China, caution is still warranted when generalizing the findings to other regions.

## 6. Conclusions

The results of this study indicate that increased concentrations of multiple air pollutants (PM_2.5_, PM_10_, SO_2_, NO_2_, and CO) are statistically associated with an elevated risk of AECOPD hospitalizations, with evidence of lagged and cumulative effects. As this is an observational study, causal inferences cannot be established. Future research incorporating multicenter designs and individual-level data is warranted to further validate the associations between air pollutants and AECOPD, as well as to elucidate the underlying mechanisms.

## Acknowledgments

This work was supported by the Hebei Provincial Health Commission (Grant No. HBZJ-2020N328). Longmei Tang has received research support from the Hebei Provincial Health Commission. The authors sincerely thank all participating hospitals and staff involved in data collection.

## Author contributions

**Conceptualization:** Jiajun Li.

**Data curation:** Yatian Liu, Xinrui Li, Jie Liu, Longmei Tang.

**Formal analysis:** Huixia Shi.

**Funding acquisition:** Longmei Tang.

**Investigation:** Huixia Shi.

**Methodology:** Dan Li, Longmei Tang.

**Software:** Jiajun Li, Boyan Li.

**Validation:** Boyan Li.

**Visualization:** Yatian Liu.

**Writing – original draft:** Huixia Shi.

**Writing – review & editing:** Dan Li, Longmei Tang.
